# *In Vivo* Evaluation of Indium-111–Labeled 800CW as a Necrosis-Avid Contrast Agent

**DOI:** 10.1007/s11307-020-01511-x

**Published:** 2020-06-08

**Authors:** Marcus C. M. Stroet, Erik de Blois, Debra C. Stuurman, Corrina M. A. de Ridder, Joost Haeck, Yann Seimbille, Laura Mezzanotte, Marion de Jong, Clemens W. G. M. Löwik, Kranthi M. Panth

**Affiliations:** 1 Erasmus MC, Department of Radiology & Nuclear Medicine, University Medical Center Rotterdam, Rotterdam, The Netherlands; 2 Erasmus MC, Department of Molecular Genetics, University Medical Center Rotterdam, Rotterdam, The Netherlands; 3 Erasmus MC, Department of Urology, University Medical Center Rotterdam, Rotterdam, The Netherlands; 4 AMIE Core Facility, Erasmus MC, Rotterdam, The Netherlands; 5 CHUV Department of Oncology, University of Lausanne, Lausanne, Switzerland

**Keywords:** Cell death, Tumor necrosis, SPECT, Cyanine dyes, Necrosis-avid contrast agents

## Abstract

**Purpose:**

Current clinical measurements for tumor treatment efficiency rely often on changes in tumor volume measured as shrinkage by CT or MRI, which become apparent after multiple lines of treatment and pose a physical and psychological burden on the patient. Detection of therapy-induced cell death in the tumor can be a fast measure for treatment efficiency. However, there are no reliable clinical tools for detection of tumor necrosis. Previously, we studied the necrosis avidity of cyanine-based fluorescent dyes, which suffered long circulation times before tumor necrosis could be imaged due to low hydrophilicity. We now present the application of radiolabeled 800CW, a commercially available cyanine with high hydrophilicity, to image tumor necrosis in a mouse model.

**Procedures:**

We conjugated 800CW to DOTA *via* a PEG linker, for labeling with single-photon emission-computed tomography isotope indium-111, yielding [^111^In]In-DOTA-PEG_4_-800CW. We then investigated specific [^111^In]In-DOTA-PEG_4_-800CW uptake by dead cells *in vitro*, using both fluorescence and radioactivity as detection modalities. Finally, we investigated [^111^In]In-DOTA-PEG_4_-800CW uptake into necrotic tumor regions of a 4T1 breast tumor model in mice.

**Results:**

We successfully prepared a precursor and developed a reliable procedure for labeling 800CW with indium-111. We detected specific [^111^In]In-DOTA-PEG_4_-800CW uptake by dead cells, using both fluorescence and radioactivity. Albeit with a tumor uptake of only 0.37%ID/g at 6 h post injection, we were able to image tumor necrosis with a tumor to background ratio of 7:4. Fluorescence and radioactivity in cryosections from the dissected tumors were colocalized with tumor necrosis, confirmed by TUNEL staining.

**Conclusions:**

[^111^In]In-DOTA-PEG_4_-800CW can be used to image tumor necrosis *in vitro* and *in vivo*. Further research will elucidate the application of [^111^In]In-DOTA-PEG_4_-800CW or other radiolabeled hydrophilic cyanines for the detection of necrosis caused by chemotherapy or other anti-cancer therapies. This can provide valuable prognostic information in treatment of solid tumors.

**Electronic supplementary material:**

The online version of this article (10.1007/s11307-020-01511-x) contains supplementary material, which is available to authorized users.

## Introduction

Necrotic tissue is typically found in pathophysiological conditions and is absent in healthy individuals. Necrosis is in most cases caused by lack of oxygen and characterized by loss of cell membrane integrity. In many pathological situations, necrosis can also be programmed like necroptosis, ferroptosis, parthanathos, and pyroptosis [1]. Most prominent examples of necrosis occur after myocardial infarcts and strokes, where the affected tissues turn necrotic due to ischemic stress [2]. In addition, solid tumors often have poor vascular quality, resulting in reduced concentration of oxygen and nutrients in the tumor center. This occurs even more in aggressively growing solid tumors as the formation of new blood vessels (angiogenesis) cannot keep up with the fast growth of the tumor. Typically, the amount of necrosis per tumor volume is a strong measurement for the aggressiveness of the tumor and used by pathologists to determine the severity of the disease [3–5]. In addition to intrinsic tumor necrosis, tumors develop necrotic tissue when affected by anti-cancer chemotherapy or radiotherapy [6].

The clinical evaluation of treatment response is normally done using CT or MRI based on the RECIST criteria, which endow clinical decision making on either treatment continuation or alternation [7]. However, these guidelines have significant shortcomings. The guidelines state that solid tumor treatment response should be determined by means of tumor size or volume reduction. However, this interpretation is prone to human error, is time consuming, and often requires multiple lines of treatment to become evident [8]. There is therefore an unmet need for novel, time-resolved criteria for early assessing the efficiency of cancer therapy treatment.

We and others have previously shown that the increase in chemotherapy or radiotherapy-induced cell death, especially necrosis, can be used as an early indicator for treatment efficiency [9–12]. Effective chemotherapy will induce significant tumor necrosis within days after a single line of treatment, due to either programmed or secondary necrosis. The latter is when massive apoptosis occurs and the few tissue macrophages present are not able to timely remove the apoptotic cells, which then become necrotic [6]. At the moment, there are no appropriate clinical tools available that allow noninvasive quantitative detection of tumor necrosis. Such a tool could be a necrosis-avid contrast agent (NACA) that specifically binds necrotic cells and can be traced in the body using imaging techniques (*e.g.,* MRI, PET, SPECT).

A frequently described NACA is hypericin, a natural product and photodynamic agent isolated from St. John’s wort, which binds to necrotic cells by an unknown mechanism. Therefore, this property has been utilized to image necrosis in various necrosis models (*e.g.,* necrotic myocardium or tumor necrosis models) by radiolabeling hypericin with iodine [13, 14]. However, hypericin has certain limitations such as poor water solubility, formation of aggregates, and difficult synthesis and it is photosensitive [15, 16]. Moreover, it can cause significant drug interactions [17].

Our group demonstrated that a certain class of near-infrared fluorescent (NIRF) cyanine dyes binds to denatured cytoplasmic proteins from cells that lost their membrane integrity. These can therefore be used for SPECT imaging of necrosis, *via* a radiometal-chelation chemistry approach [9, 11]. However, the HQ class of cyanines that we have used was thermally unstable and henceforth was conjugated with the non-linear DTPA chelator. DTPA allows indium-111 chelation at room temperature, but other DTPA-radiometal complexes have low *in vivo* stability [18]. Moreover, HQ-class cyanines are hydrophobic, leading to long circulation times before reaching the target tissue. This is a limiting factor for clinical translation since it is preferable to image shortly after administration.

Here, we describe radiolabeling of 800CW, a cyanine with improved water solubility and NIRF properties compared to HQ5 (785-nm excitation, 800-nm emission of 800CW; and 683-nm excitation, 701-nm emission of HQ5) [9]. The more redshifted wavelength of 800CW, compared to HQ4 and HQ5, has deeper tissue penetration of up to 1–2 cm and is henceforth favorable for *in vivo* applications, especially in small animals or in superficial tissues like skin, breast, and head and neck in humans [19]. However, for whole body imaging, a more quantitative imaging technology is needed like nuclear imaging using PET or SPECT. For this reason, we conjugated 800CW, *via* a polyethylene glycol (PEG) linker to DOTA, a widely applied chelator in the clinic for radiolabeling with SPECT-isotope indium-111 [18]. We first characterized and determined the purity of DOTA-PEG_4_-800CW (**1**) by HPLC and mass spectrometry. Thereafter, a reliable radiolabeling procedure was developed and we tested *in vitro* necrosis-avidity of DOTA-PEG_4_-800CW with and without indium-111 labeling. After validation, indium-111–labeled **1** ([^111^In]In-DOTA-PEG_4_-800CW, from here referred to as [^111^In]**1**) was tested *in vivo* in the mouse 4T1 breast cancer model that spontaneously develops tumor necrosis.

## Materials and Methods

### Materials

Reagents were purchased from Sigma-Aldrich (St. Louis, MO, USA) unless stated otherwise. Solvents were purchased from Honeywell Riedel-de-Haën™ (Seelze, Germany). 800CW-*N*-hydroxysuccinimide ester (**2**) was purchased from Westburg BV (Leusden, The Netherlands). DOTA-PEG_4_-NH_2_ (**3**) was purchased from Chematech (Dijon, France). [^111^In]InCl_3_ from Mallinckrodt BV (Petten, The Netherlands). 4T1-Luc2 cells were purchased from PerkinElmer (Boston, MA, USA). Cell culture media was obtained from Sigma-Aldrich (St. Louis, MO, USA) or Gibco Life Technologies (Waltham, MA, USA). ESI-MS analysis was performed on a TSQ Quantum Ultra system equipped with a Surveyor Autosampler Plus and MS Pump Plus from Thermo Fisher Scientific (San Jose, CA, USA). HPLC analysis was performed on a system from Waters (Milford, MA, USA) with a quaternary pump, a 2998 PDA detector, a radio detector consisting of a NaI crystal detector with a Canberra Osprey-DTB, and dedicated software. Solvent A was water + 0.1 % *v*/v TFA, and solvent B was ACN + 0.1 % v/v TFA. HPLC method A is Gemini C_18_ semi-preparative column (10 × 250 mm, 5 μm, Waters) and a gradient profile of 0–1 min 20 % B in A, 1–25 min to 30 % B at a flow rate of 3.0 ml/min. HPLC method B is Symmetry C_18_ analytical column (4.6 × 250 mm, 5 μm, Waters) and a gradient profile of 0–1 min 10 % B, 1–20 min towards 60 % B at a flow rate of 1.0 ml/min. HPLC method C is Symmetry C_18_ analytical column (4.6 × 250 mm, 5 μm, Waters) and a gradient profile of 0–1 min 22 % B, 1–20 min towards 51 % B (solvent A was 0.2 M Tris-HCl buffer pH 8.5 + 10 % MeOH and solvent B was MeOH) at a flow rate of 1.0 ml/min. Autoradiography was performed using super resolution phosphor screens and a Cyclone® Plus system (Perkin Elmer, Waltham, MA, USA). NIRF imaging was performed on an Odyssey flatbed scanner system (800 nm channel, laser intensity 5.0; Li-Cor, Lincoln, NE, USA). Bioluminescence imaging (BLI) was performed in an IVIS-Spectrum imager (Perkin Elmer). SPECT/CT images were obtained with a VECTor-5, equipped with a high-sensitivity 3.0-mm pinhole collimator (MILabs, Utrecht, The Netherlands). The mages were analyzed with Pi-Mod (version 3.901) and visualized in VivoQuant (version 2.50, patch 3). Activity was accurately quantified on a Wizard 3″, 1480 γ-counter (Perkin Elmer). Histochemical dead cell staining was performed using the DeadEnd™ Colorimetric TUNEL System (Promega, Madison, WI, USA) and subsequent imaging on a NanoZoomer 2.0HT digital slide scanner (Hamamatsu, Hamamatsu City, Japan).

### Synthesis DOTA-PEG_4_-800CW (**1**)

Compound **2** (5.0 mg, 4.4 μmol) was dissolved in DMSO (50 μl) and added to a mixture of DOTA-PEG_4_-NH_2_ (**3**, 3.1 mg, 3.8 μmol) and DiPEA (1.78 μl, 10.1 μmol) in DMSO (200 μl). The resulting solution was stirred overnight at room temperature. Subsequently, the mixture was directly purified over semi-preparative HPLC, method A (t_R_ 14 min), yielding a green solid (4.1 mg, 55 %). The purity was > 99 % as determined by HPLC, method B (t_R_ 10.1 min, Supplementary data 1). ESI-MS: *m/z* calculated for C_72_H_103_N_8_O_25_S_4_^+^ was 1607.59. *m/z* found was 802.67 [M-3H]^2−^; 534.75 [M-4H]^3−^.

### Synthesis Reference Compound in-DOTA-PEG_4_-800CW (^Nat.^In-**1**)

Compound **1** (430 μg, 250 nmol) was added into a conical vial, and quenchers were added to mimic reaction conditions of radiolabeling (gentisic acid and sodium ascorbate with a final concentration of 3.5 mM). The reaction was buffered to pH 4–4.5 with sodium acetate (53.6 μl, 2.5 M) and diluted with Milli-Q water to a final reaction volume of 135 μl. Then, InCl_3_ (375 nmol, native indium ICP standard in 0.05 M HCl) was added, and the sealed vial was heated to 70 °C for 30 min. The vial was allowed to cool to room temperature and DTPA (4 mM, 5 μl) was added to complex remaining indium. The conversion yield was > 99 % as determined by HPLC, method B (t_R_ 9.5 min, Supplementary data 2). ESI-MS: *m/z* calculated for C_72_H_100_N_8_O_25_S_4_In^+^ was 1719.47. *m*/*z* found was 860.19 [M+H]^2+^.

### Radiolabeling

Into a conical vial, gentisic acid and sodium ascorbate (both 50 mM in 10 μl) were added to **1** (1.4/14/70 μg; 0.8/8.0/40 nmol; for low, medium, or high dose, respectively) to prevent radiolysis [20]. The reaction was buffered to pH 4–4.5 with sodium acetate (1 μl, 2.5 M) and diluted with Milli-Q water for a final reaction volume of 135 μl. Then, [^111^In]InCl_3_ (80 MBq) was added and the sealed vial was heated to 70 °C for 30 min. The vial was cooled to room temperature for 5 min, and DTPA (4 mM, 5 μl) was added. Radiochemical purity (RCP) of [^111^In]**1** was > 95 % as determined by HPLC, method C (t_R_ 14.1 min). Radiochemical yield was determined by an instant thin-layer chromatography (iTLC-SG paper, Aligant, eluent: 10 % *w*/*v* NH_4_Ac to MeOH 9:1, [^111^In]In-DTPA eluted to top of paper and [^111^In]**1** remained at the baseline).

### *In Vitro* Dead/Alive Cell Uptake Assay Fluorescence

4T1-Luc2 cells were cultured in RPMI-1640 medium containing 10 % fetal bovine serum and 1 % penicillin at 37 °C under a humidified atmosphere with 5 % CO_2_. The cells were seeded in a black 96-well plate with transparent bottom (typically 1.0 × 10^4^ cells per well) and grown until 70–80 % confluency. Then, from half of the wells, the medium was removed, the cells were killed with EtOH (4 μl, 70 %) and washed once with PBS. The live cells in the other half of the wells were washed with PBS, and subsequently, all cells were incubated for 15 min with or without 800CW or it conjugates (100 nM in culture medium) as indicated. The cells were washed thrice with PBS, fixated with 4 % formalin, and the whole plate was imaged on the Odyssey. This experiment was performed in triplicate.

### *In Vitro* Dead/Alive Cell Uptake Assay Radioactivity

4T1-Luc2 cells were seeded in two 12-well plates (1.0 × 10^5^ cells per well) and grown until 70–80 % confluency. After incubation, the medium was removed from half of the wells, after which the cells were killed with EtOH (50 μl, 70 %) and washed once with PBS. The live cells were washed with PBS, and subsequently, all cells were incubated for 1 h at 37 °C with or without the radiotracers (3.3 MBq/nmol, 100 nM in 400 μl culture medium). Then, the cells of one plate were washed twice with PBS, detached with 1.0 M NaOH, and collected in tubes for γ-counting. The cells of the other plate were washed twice with PBS; the whole plate was imaged on a phosphor screen (2 h) and the Odyssey. This experiment was performed in triplicate.

### Animals

All animal experiments were approved by the Animal Welfare Committee and conducted in accordance with accepted guidelines. Female BALB/cAnNRj-nude mice (6 to 8 weeks old) were housed in ventilated cages in groups of four to six mice and were provided standard laboratory animal food pellets and water *ad libitum*. A week after arrival, 1.0 × 10^4^ 4T1-Luc2 cells suspended in 15 μl matrigel: PBS (1:1) were injected bilaterally on the shoulders. Tumor growth was monitored every 3 days with a caliper. After 2 weeks, mice were injected with d-luciferin (150 mg/kg, i.p. injection). Ten minutes later, BLI was performed for 30 s with an open filter under isoflurane anesthesia (4 % induction, 1.5 to 2 % maintenance in 100 % O_2)_. All groups consisted of three mice with bilateral tumors.

### SPECT Imaging

Tumor-bearing mice received an intravenous injection of [^111^In]**1** (20 MBq, labeled to 0.2, 2, or 10 nmol, 200 μl PBS) in the tail vein. At 6 and 24 h post injection (h.p.i.), SPECT/CT imaging was performed under isoflurane anesthesia, while the body temperature was maintained constant. Static images of the 5-cm axial field of view were obtained over a total scan time of 28 min and 20 s, followed by a 2-min full body CT. Dynamic scans were obtained over a total duration of 1 h with 30 timeframes, directly after injection of [^111^In]**1**.

Acquired images were reconstructed using SR-OSEM with 9 iterations and 128 subsets on a 36 × 36 × 35 mm matrix with 0.80 × 0.80 mm isotropic voxels. The images were further analyzed in Pi-Mod and VivoQuant. Regions of interest were manually drawn around the tumors, heart, and muscle. Subsequently, the percentage of injected dose (%ID) and the tumor to background ratio (TBR) were determined.

### Urine Analysis

Excreted urine was collected at 1 h.p.i. and frozen in liquid nitrogen. The urine was then thawed, precipitated with an equal volume of ACN, centrifuged at rcf: 16100×*g* for 15 min, and the supernatant was analyzed by radio-HPLC. As a control for the processing, 1 MBq of the NACA in PBS was treated in a similar way as the urine.

### *Ex vivo* Analysis

After imaging, the blood, tumors, skin, pancreas, liver, spleen, small intestines, colon, ovaries, kidneys, lungs, heart, muscle, bone, lymph nodes, and brain were collected and weighted, and activity was accurately quantified in a γ-counter. After counting, the tumors were frozen in liquid nitrogen and adjacent 10-μm cryosections of tumor centers were prepared for autoradiography, NIRF imaging, and TUNEL staining.

### Statistics

All data are expressed as the mean ± standard deviation (SD). Significance was determined with F-test and two-tailed *t* test in Microsoft Excel 2010.

## Results

### Synthesis and Radiolabeling

**Fig. 1:**
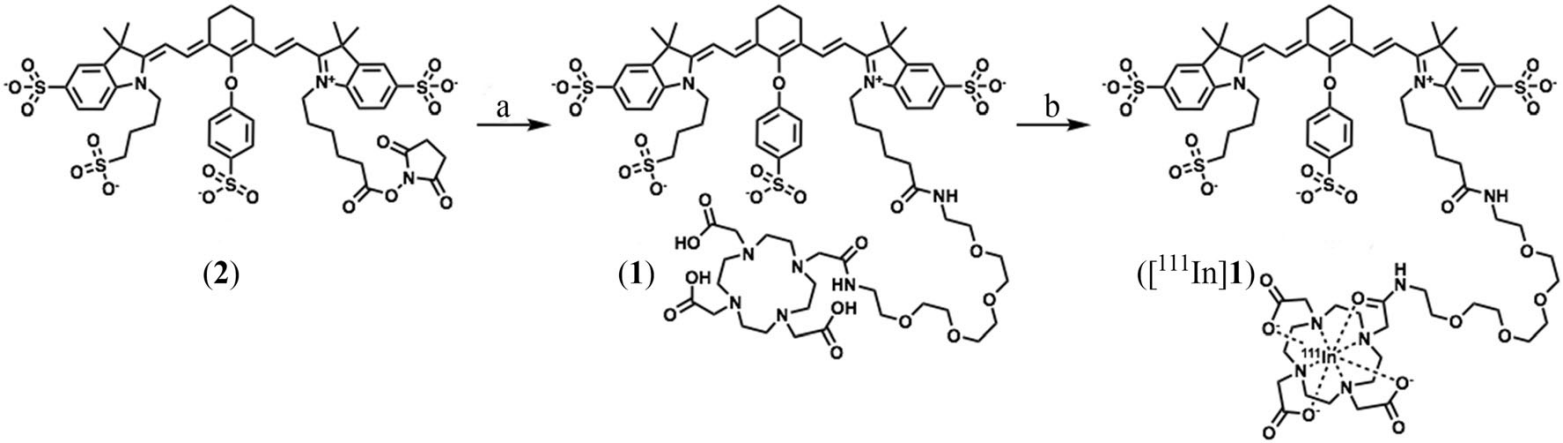
Synthesis of DOTA-PEG_4_-800CW. **a** DOTA-PEG_4_-NH_2_ (**3**), DiPEA, DMSO, rt., 16 h, yield 55 %. **b** NaAc, gentisic acid, sodium ascorbate, H_2_O, 70 °C, 30 min, RCP > 98 %.

Precursor **1** was successfully synthesized *via* an amide coupling reaction between **2** and **3** (Fig. 1). After purification, 4.1 mg (55 % yield) of precursor was isolated as a green solid, with a purity of > 99 % (HPLC), and was stored in titrated aliquots of 15.6 nmol at − 20 °C.

The RCP and RCY of [^111^In]**1** were > 95 % with molar activities achieved up to 200 MBq/nmol. The antioxidant gentisic acid and ascorbic acid in the reaction mixture stabilized radiochemical purity. After 2 days at room temperature (10 MBq/nmol/200 μl), RCP was > 95 % (Supplementary data 3). All *in vitro* and *in vivo* experiments were executed within 3 h after radiolabeling.

### *In Vitro* Dead/Alive Cell Uptake

**Fig. 2:**
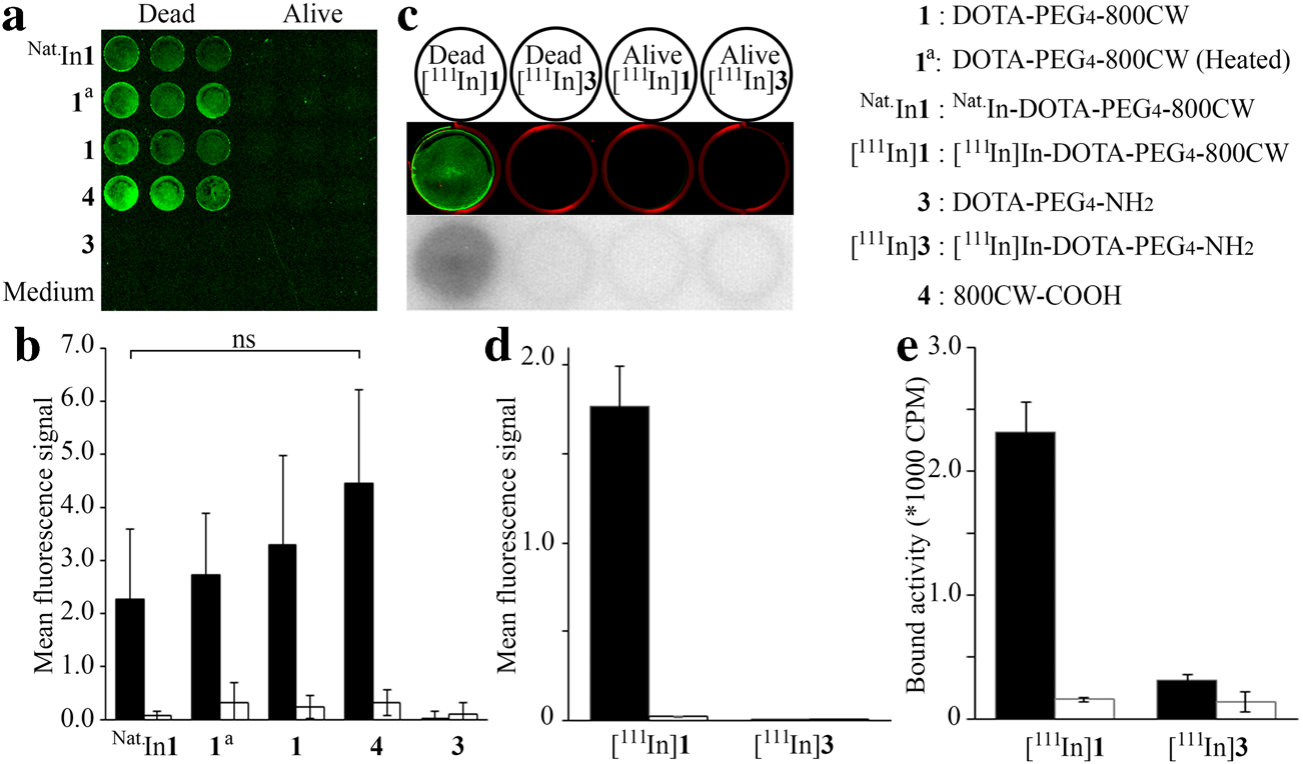
*In vitro* dead cell binding of 800CW. **a** Dead or alive 4T1-Luc2 cells in a 96-well plate, treated with either ^Nat.^In-**1**, **1**^**a**^ (heated at 70 °C for 30 min), **1**, **4**, **3**, or culture medium. **b** Fluorescent signal from **a**, black bars indicate signal from dead cells and white bars indicate signal from alive cells (*n* = 9). **c** Dead or alive 4T1-Luc2 cells in a 12-well plate, treated with either [^111^In]**1** or [^111^In]**3**. The 800-nm channel is depicted in green, and the 700-nm channel is depicted in red, and below the corresponding autoradiography signal. **d**, **e** Fluorescent signal (**d**) and bound activity (**e**) from wells in **c.** ns = no significant difference.

We further examined the effect of the metal chelation and indium labeling on the necrosis avidity of 800CW in dead or alive 4T1-Luc2 cells. Based on fluorescent signal from the cells after washing, the uptake of 800CW and its conjugates was determined (Fig. 2a).

The following conditions were tested: In-DOTA-PEG_4_-800CW (^Nat.^In-**1**), labeled with non-radioactive indium; DOTA-PEG_4_-800CW which was heated at 70 °C for 30 min (**1**^**a**^) [20], untreated **1**, 800CW-carboxylate (**4**), DOTA-PEG_4_-NH_2_ (**3**) as a negative control, and medium for background subtraction. The experiment was performed in triplicate (Fig. 2a). Based on the fluorescent signal, we found that neither the procedures required for radiolabeling nor the indium complexation of 800CW had a significant effect on uptake by dead cells (Fig. 2b).

Later, we determined the [^111^In]**1** uptake by dead cells and used [^111^In]In-DOTA-PEG_4_-NH_2_ ([^111^In]**3**) as a negative control. The wells were washed, and the whole plate was imaged on a phosphor screen for visualization of the radioactivity and on the Odyssey (Fig. 2c) for quantification of the fluorescence. The fluorescent signal from the dead cells was a 67-fold stronger than that from the live cells (Fig. 2d). As expected, no fluorescence signal was observed from the [^111^In]**3**-treated wells. Simultaneously, a second plate was prepared, from which the cells were collected from the wells after incubation and washing. The radioactivity was measured in a γ-counter for accurate quantification of the radioactivity uptake (Fig. 2e). There was no uptake of [^111^In]**3** by dead or alive cells, however a 23-fold higher uptake of [^111^In]**1** by dead cells as compared to live cells.

### SPECT Imaging and *Ex Vivo* Analysis

[^111^In]**1** was administrated in female nude mice inoculated with 4T1-Luc2 breast cancer cells, which develop rapidly growing tumors with spontaneous necrotic cores. The necrotic core was first confirmed by BLI since 4T1-Luc2 cells express luciferase. As a result, necrotic tumors showed a ring-shaped BLI signal from their viable outer rim and a much lower signal from the center (Supplementary data 4) [9].

In the first *in vivo* experiment, we evaluated the dose range in which the tumors could be imaged. Therefore, three doses of **1** (*i.e.,* 0.2 nmol, 2.0 nmol, and 10 nmol; from here referred to as low, medium, and high dose) with a constant amount of radioactivity (*i.e.,* 20 MBq) per injection were tested. At 6 and 24 h.p.i., SPECT/CT imaging was performed. Then, the animals were euthanized and the organs and tumors were collected for biodistribution and histological analysis.

**Fig. 3:**
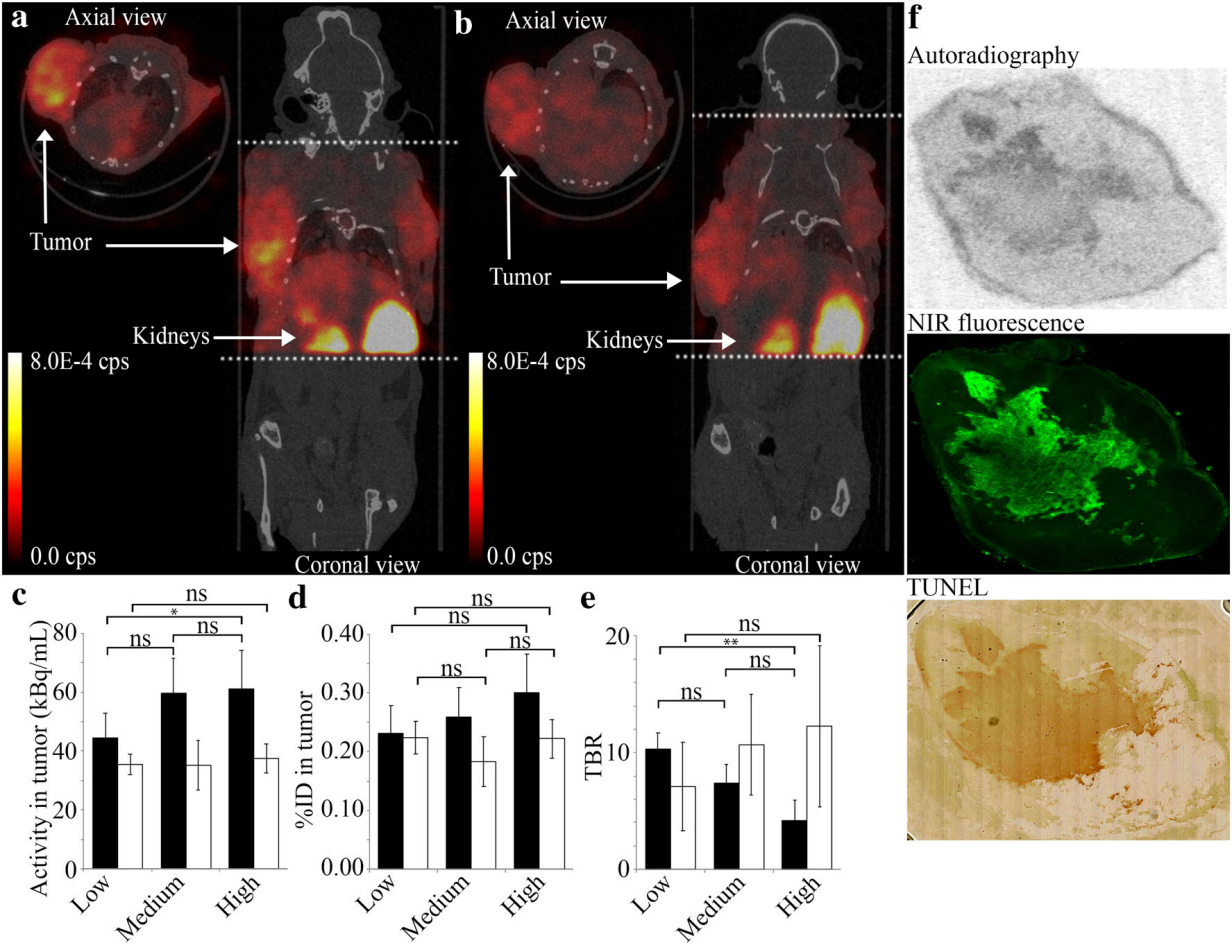
SPECT/CT imaging with [^111^In]**1**. **a**, **b** Representative SPECT/CT image of a mouse injected with [^111^In]**1** at 6 h.p.i. (**a**) and 24 h.p.i. (**b**). The dotted lines indicate field of view of SPECT images. **c** Concentration of activity in tumor ROIs from SPECT images in kBq/ml, *n* = 6. **d** Activity in tumor, normalized injected dose tumor uptake %ID (*n* = 6). **e** TBR (tumor to heart) from SPECT signal, *n* = 6. **f** Adjacent cryosections from a dissected tumor. From top to bottom: autoradiography, NIRF imaging, and TUNEL staining. * for *p* < 0.05; ** for *p* < 0.01.

**Fig. 4:**
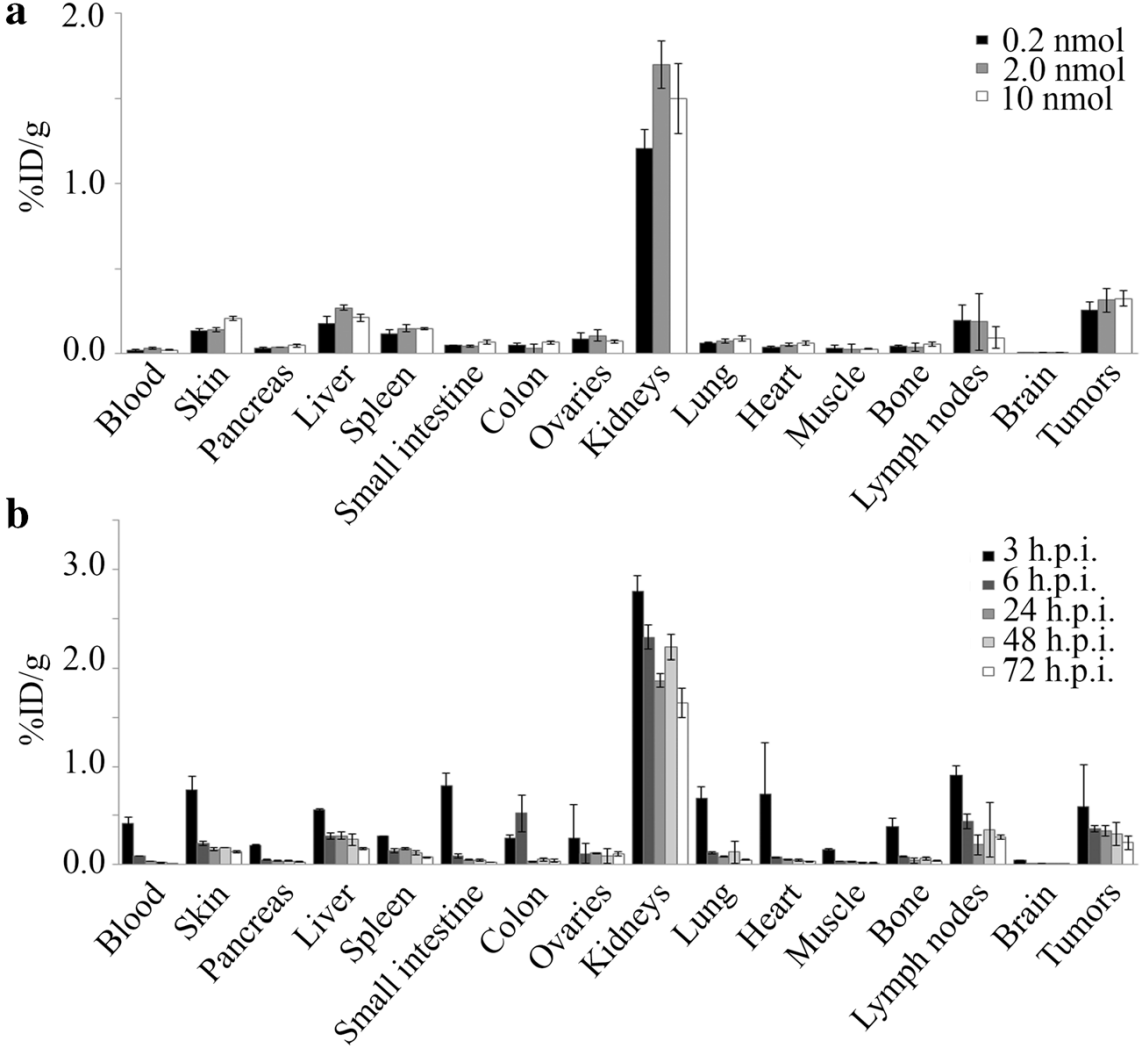
Biodistribution of [^111^In]**1**. **a** Biodistribution at 24 h.p.i. of three different doses of [^111^In]**1** (*n* = 3). **b** Biodistribution of [^111^In]**1** (2 nmol, 20 MBq) at five different time points after injection (*n* = 3).

The uptake in the tumors and kidneys were clearly visible on SPECT images at 6 and 24 h.p.i (Fig. 3 a and b). The SPECT signal from the tumors was quantified and showed no significant difference in the total counts between high and medium dose at 6 h.p.i. On the other hand, the total counts were significantly lower in the low-dose group (44 kBq/ml) compared to the high-dose group (61 kBq/ml, *P* = 0.03, Fig. 3c). However, no major differences were observed in %ID between the three doses at 6 h.p.i. (Fig. 3d). At 24 h.p.i, no significant differences in either total counts or %ID was seen between the three doses. Similarly, the biodistribution studies also showed no significant differences in %ID/g between the three dose groups at 24 h.p.i. (Fig. 4a), confirming these results. Even though the overall uptake in the tumors was low, the tumors were clearly visible on SPECT images due to high TBRs (Fig. 3e). The TBR for the low-dose group was the highest (10:3), followed by medium-dose group (7:4), and high-dose group (4:2, *P* = 0.087). At 24 h.p.i., however, there were no significant differences between the three doses (Fig. 3c–e). The uptake of the NACA into the necrotic regions of the extracted tumor was confirmed by autoradiography, NIRF imaging, and TUNEL staining of the cryosections (Fig. 3f).

Next, we evaluated the optimal time-point for imaging. The NACA uptake in %ID/g was determined at 3, 6, 24, 48, and 72 h.p.i. At each time-point, mice were euthanized and the organs were collected to determine the biodistribution of the NACA (Fig. 4). The tumor uptake did not increase over time, and no significant differences were observed between time-points. However, it can be noted that the NACA is rapidly excreted from the body with 3%ID/g left in the kidneys after 3 h. As a result, the tumor to blood ratios increased over time, up to 29:7 at 72 h.p.i. (Supplementary data 5).

**Fig. 5:**
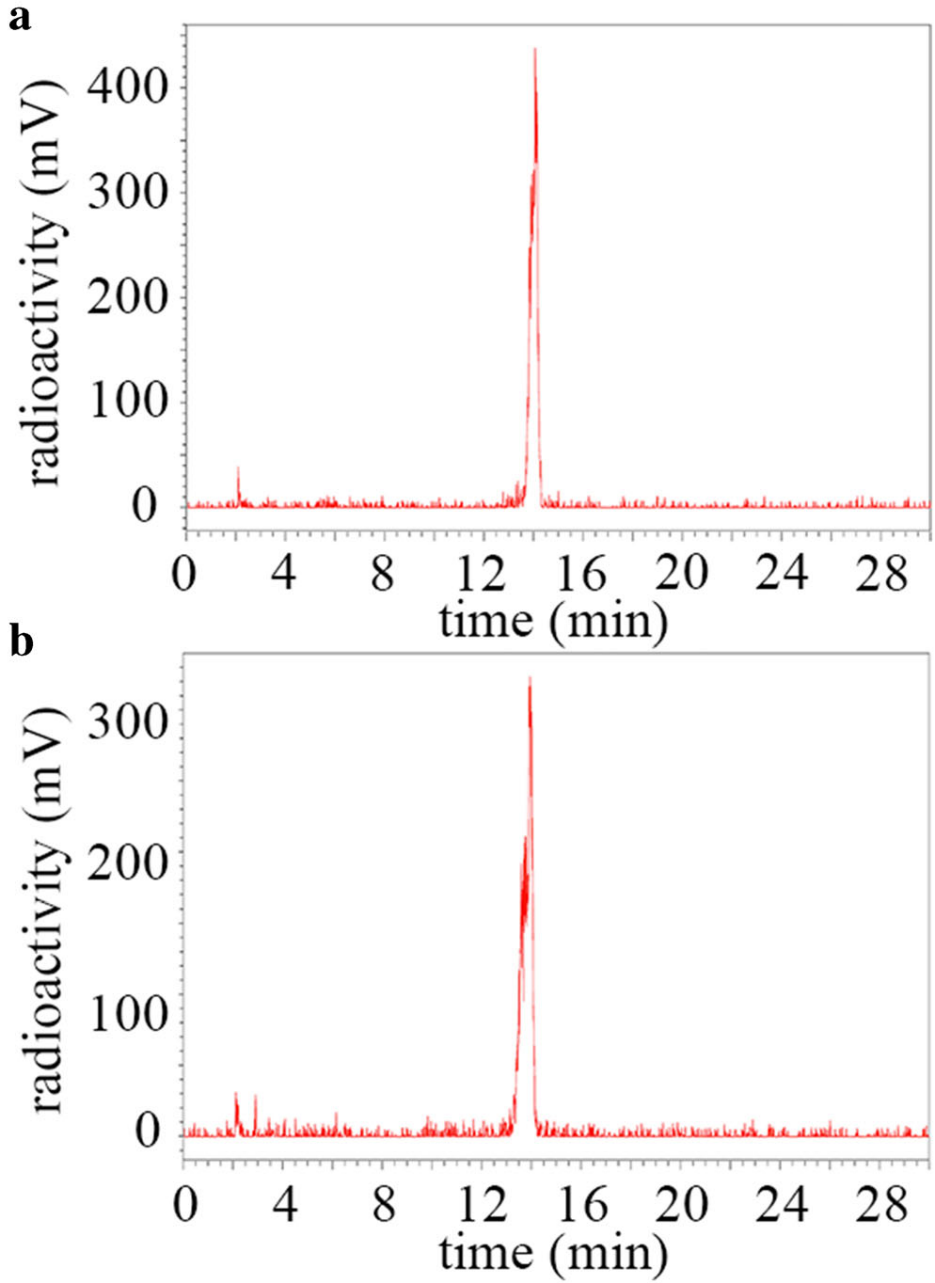
**a** HPLC chromatogram of [^111^In]**1** from PBS. **b** HPLC chromatogram of urine from mouse at 1 h.p.i.

We performed dynamic scans of the first hour after injection of the NACA, in which we observed renal clearance of most of the NACA in the first recorded frame recorded 0–5 min post injection (Supplementary data 6). At 1 h.p.i., urine was collected and analyzed by radio-HPLC (method C, 0.2 MBq injected, recovery > 95 %). We observed that > 98 % of the activity eluted at 14 min indicating that the NACA was excreted intact (Fig. 5).

## Discussion

In a previous publication using the cyanines HQ4, HQ5, and 800CW, we demonstrated that we can image tumor necrosis in mice using whole body NIRF imaging techniques [9]. For clinical translation, we conjugated HQ4 with DTPA and radiolabeled it with indium-111 for SPECT imaging and showed that tumor necrosis could be imaged with good TBRs [11, 21]. However, we foresaw that HQ cyanines are sub-optimal for clinical translation because they are lipophilic and hence are cleared by the hepatic-intestinal route and showed long blood circulation. Therefore, > 24 h were required for a good tumor to background ratio. This ratio is in contrast to 800CW, which is a highly hydrophilic cyanine that is cleared by the kidneys and has a good TBR after 3–6 h [9]. Especially when using radiotracers, rapid clearance is preferred to minimize radioactive exposure to the organs. Therefore, in this study, we determined the use of 800CW as a necrosis-specific probe for SPECT by radiolabeling with indium-111. In order to do so, we conjugated 800CW with DOTA, one of the most used chelators in the clinic. We proceeded to test the necrosis avidity *in vitro* with our in-house–developed dead cell assay. Based on the fluorescent signal, the 800CW uptake by dead cells is not significantly affected by conjugation with DOTA *via* a PEG_4_ linker and subsequent chelation of indium. This is in line with the previous findings that 800CW could image necrosis when conjugated with small molecule probes [9]. Moreover, we found that 800CW was not degraded by the heating, required for radiolabeling.

From the *in vivo* imaging experiments, it was evident that there was no significant difference in tumor uptake between the tested doses. However, the TBR was greater in the group that received a low dose (10:3) compared to medium dose (7:4) and high dose (4:2). This confirms that even at low doses it is feasible to image necrosis with 800CW. However, we wanted to use the fluorescent signal, which could not be detected at the low dose, for further confirmation. Hence, the medium dose was selected for further experiments since a clear fluorescent signal was obtained.

In order to increase the dose, while maintaining the amount of activity injected (20 MBq) constant, the molar activity was required to be reduced. The molar activity is an important parameter that should be considered with radiopharmaceuticals that target receptors or proteins since there is only a limited number of proteins available in the targeted tissue. Decreasing the molar activity can result in a saturation of the target protein, rendering a lower activity uptake in the targeted tissue [22]. This effect is not observed in the case of necrosis-avid cyanines. This could be due to their binding to denatured cytosolic proteins, which are highly abundant and unlikely to saturate [9].

Necrosis-targeting probes, such as hypericin, have been successfully shown to target necrosis and also to deliver local radionuclide therapy in the center of necrotic tumors [14, 16]. However, certain limitations such as difficult synthesis, poor water solubility, and toxicity effects have been reported for these probes [15–17]. NIR cyanines like 800CW could be an interesting starting point for developing novel clinical tools, since they have been applied in ample preclinical and clinical applications. For instance, ICG, a serum albumin-binding NIR cyanine, is the current golden standard for visualizing tissue perfusion in the emerging field of fluorescence-guided surgery [23]. Likewise, 800CW has been applied in several clinical studies when conjugated to a tumor-targeting molecule without any reports of toxicity induced by the cyanine itself [24, 25].

As compared to [^111^In]In-DTPA-HQ4, [^111^In]**1** has a faster clearance from the blood, which is mainly accommodated by the renal system. This is most likely because of the higher water solubility of 800CW. As a result, the background signal dropped rapidly and SPECT/CT imaging could be performed at 6 h.p.i. With [^111^In]In-DTPA-HQ4, imaging could only be performed 24 h.p.i. onwards. It should also be noted that the tumor uptake of [^111^In]**1** was lower than that of [^111^In]In-DTPA-HQ4 but sufficient for imaging the tumors [21].

Due to the rapid biokinetics, shorter lived radioisotopes would be more suitable for imaging with **1**. For instance, **1** could be labeled with PET-radionuclide gallium-68 [26].

## Conclusions

We demonstrated that [^111^In]**1** can be used to image tumor necrosis in mice. It is rapidly excreted from the body, and the tumors can be imaged at high contrast at early time points after injection. We showed the uptake of [^111^In]**1** into spontaneous developing tumor necrosis in a mouse model with 4T1-Luc2 breast cancer. Future studies should elucidate whether [^111^In]**1** can be used to image tumor necrosis, which is induced by chemotherapy or other anti-cancer therapies.

## Electronic supplementary material

ESM 1 (DOCX 21292 kb)

## References

[1] Galluzzi L, Vitale I, Aaronson SA, Abrams JM, Adam D, Agostinis P, Alnemri ES, Altucci L, Amelio I, Andrews DW, Annicchiarico-Petruzzelli M, Antonov AV, Arama E, Baehrecke EH, Barlev NA, Bazan NG, Bernassola F, Bertrand MJM, Bianchi K, Blagosklonny MV, Blomgren K, Borner C, Boya P, Brenner C, Campanella M, Candi E, Carmona-Gutierrez D, Cecconi F, Chan FKM, Chandel NS, Cheng EH, Chipuk JE, Cidlowski JA, Ciechanover A, Cohen GM, Conrad M, Cubillos-Ruiz JR, Czabotar PE, D’Angiolella V, Dawson TM, Dawson VL, de Laurenzi V, de Maria R, Debatin KM, DeBerardinis RJ, Deshmukh M, di Daniele N, di Virgilio F, Dixit VM, Dixon SJ, Duckett CS, Dynlacht BD, el-Deiry WS, Elrod JW, Fimia GM, Fulda S, García-Sáez AJ, Garg AD, Garrido C, Gavathiotis E, Golstein P, Gottlieb E, Green DR, Greene LA, Gronemeyer H, Gross A, Hajnoczky G, Hardwick JM, Harris IS, Hengartner MO, Hetz C, Ichijo H, Jäättelä M, Joseph B, Jost PJ, Juin PP, Kaiser WJ, Karin M, Kaufmann T, Kepp O, Kimchi A, Kitsis RN, Klionsky DJ, Knight RA, Kumar S, Lee SW, Lemasters JJ, Levine B, Linkermann A, Lipton SA, Lockshin RA, López-Otín C, Lowe SW, Luedde T, Lugli E, MacFarlane M, Madeo F, Malewicz M, Malorni W, Manic G, Marine JC, Martin SJ, Martinou JC, Medema JP, Mehlen P, Meier P, Melino S, Miao EA, Molkentin JD, Moll UM, Muñoz-Pinedo C, Nagata S, Nuñez G, Oberst A, Oren M, Overholtzer M, Pagano M, Panaretakis T, Pasparakis M, Penninger JM, Pereira DM, Pervaiz S, Peter ME, Piacentini M, Pinton P, Prehn JHM, Puthalakath H, Rabinovich GA, Rehm M, Rizzuto R, Rodrigues CMP, Rubinsztein DC, Rudel T, Ryan KM, Sayan E, Scorrano L, Shao F, Shi Y, Silke J, Simon HU, Sistigu A, Stockwell BR, Strasser A, Szabadkai G, Tait SWG, Tang D, Tavernarakis N, Thorburn A, Tsujimoto Y, Turk B, vanden Berghe T, Vandenabeele P, Vander Heiden MG, Villunger A, Virgin HW, Vousden KH, Vucic D, Wagner EF, Walczak H, Wallach D, Wang Y, Wells JA, Wood W, Yuan J, Zakeri Z, Zhivotovsky B, Zitvogel L, Melino G, Kroemer G (2018). Molecular mechanisms of cell death: recommendations of the nomenclature committee on cell death 2018. Cell Death Differ.

[2] Malhotra R, Lin Z, Vincenz C, Brosius FC (2001). Hypoxia induced apoptosis via two independent pathways in Jurkat cells: differential regulation by glucose. Am J Physiol Cell Physiol.

[3] Lee SY, Ju MK, Jeon HM et al (2018) Regulation of tumor progression by programmed necrosis. Oxid Med Cell Longev 2018:3537471. 10.1155/2018/3537471 10.1155/2018/3537471 PMC5831895 29636841

[4] Bredholt G, Mannelqvist M, Stefansson IM, Birkeland E, Bø TH, Øyan AM, Trovik J, Kalland KH, Jonassen I, Salvesen HB, Wik E, Akslen LA (2015). Tumor necrosis is an important hallmark of aggressive endometrial cancer and associates with hypoxia, angiogenesis and inflammation responses. Oncotarget.

[5] Richards CH, Mohammed Z, Qayyum T, Horgan PG, McMillan DC (2011). The prognostic value of histological tumor necrosis in solid organ malignant disease: a systematic review. Future Oncol.

[6] Silva MT (2010). Secondary necrosis: the natural outcome of the complete apoptotic program. FEBS Lett.

[7] Eisenhauer EA, Therasse P, Bogaerts J, Schwartz LH, Sargent D, Ford R, Dancey J, Arbuck S, Gwyther S, Mooney M, Rubinstein L, Shankar L, Dodd L, Kaplan R, Lacombe D, Verweij J (2009). New response evaluation criteria in solid tumours: Revised RECIST guideline (version 1.1). Eur J Cancer.

[8] Abdulla S, Saada J, Johnson G, Jefferies S, Ajithkumar T (2015). Tumour progression or pseudoprogression? A review of post-treatment radiological appearances of glioblastoma. Clin Radiol.

[9] Xie B, Stammes MA, van Driel PBAA, Cruz LJ, Knol-Blankevoort VT, Löwik MAM, Mezzanotte L, Que I, Chan A, van den Wijngaard JPHM, Siebes M, Gottschalk S, Razansky D, Ntziachristos V, Keereweer S, Horobin RW, Hoehn M, Kaijzel EL, van Beek ER, Snoeks TJA, Löwik CWGM (2015). Necrosis avid near infrared fluorescent cyanines for imaging cell death and their use to monitor therapeutic efficacy in mouse tumor models. Oncotarget.

[10] Xie B, Tomaszewski MR, Neves AA, Ros S, Hu DE, McGuire S, Mullins SR, Tice D, Sainson RCA, Bohndiek SE, Wilkinson RW, Brindle KM (2017). Optoacoustic detection of early therapy-induced tumor cell death using a targeted imaging agent. Clin Cancer Res.

[11] Stammes MA, Maeda A, Bu J (2016). The necrosis-avid small molecule HQ4-DTPA as a multimodal imaging agent for monitoring radiation therapy-induced tumor cell death. Front Oncol.

[12] Salah S, Lewin J, Amir E, Abdul Razak A (2018). Tumor necrosis and clinical outcomes following neoadjuvant therapy in soft tissue sarcoma: a systematic review and meta-analysis. Cancer Treat Rev.

[13] Cona M, Li J, Feng Y, Chen F, Verbruggen A, Witte P, Oyen R, Ni Y (2014). Targetability and biodistribution of radioiodinated hypericin: comparison between microdosing and carrier-added preparations. Anti Cancer Agents Med Chem.

[14] Abma E, Peremans K, De Vos F (2018). Biodistribution and tolerance of intravenous iodine-131-labelled hypericin in healthy dogs. Vet Comp Oncol.

[15] Jiang B, Wang J, Ni Y, Chen F (2013). Necrosis avidity: a newly discovered feature of hypericin and its preclinical applications in necrosis imaging. Theranostics.

[16] Ji Y, Zhan Y, Jiang C, Jiang X, Gao M, Liu W, Li Y, Wang J, Wang Q, Cona MM, Yao N, Wang X, Fang Z, Yin Z, Zhang J, Sun Z, Ni Y (2014). Improvement of solubility and targetability of radioiodinated hypericin by using sodium cholate based solvent in rat models of necrosis. J Drug Target.

[17] Henderson L, Yue QY, Bergquist C, Gerden B, Arlett P (2002). St John’s wort (Hypericum perforatum): drug interactions and clinical outcomes. Br J Clin Pharmacol.

[18] Camera L, Kinuya S, Garmestani K, Wu C, Brechbiel MW, Pai LH, McMurry T, Gansow OA, Pastan I, Paik CH (1994). Evaluation of the serum stability and in vivo biodistribution of CHX-DTPA and other ligands for yttrium labeling of monoclonal antibodies. J Nucl Med.

[19] Zhao J, Chen J, Ma S, Liu Q, Huang L, Chen X, Lou K, Wang W (2018). Recent developments in multimodality fluorescence imaging probes. Acta Pharm Sin B.

[20] Hernandez R, Heskamp S, Rijpkema M, Bos DL, Goldenberg DM, McBride WJ, Morgenstern A, Bruchertseifer F, Cai W, Boerman OC (2017). Preventing radiobleaching of cyanine fluorophores enhances stability of nuclear/NIRF multimodality imaging agents. Theranostics.

[21] Stammes MA, Knol-Blankevoort VT, Cruz LJ, Feitsma HRIJ, Mezzanotte L, Cordfunke RA, Sinisi R, Dubikovskaya EA, Maeda A, DaCosta RS, Bierau K, Chan A, Kaijzel EL, Snoeks TJA, van Beek ER, Löwik CWGM (2016). Pre-clinical evaluation of a cyanine-based SPECT probe for multimodal tumor necrosis imaging. Mol Imaging Biol.

[22] Keller T, López-Picón FR, Krzyczmonik A, Forsback S, Takkinen JS, Rajander J, Teperi S, Dollé F, Rinne JO, Haaparanta-Solin M, Solin O (2019). Comparison of high and low molar activity TSPO tracer [(18)F]F-DPA in a mouse model of Alzheimer’s disease. J Cereb Blood Flow Metab.

[23] van Manen L, Handgraaf HJM, Diana M, Dijkstra J, Ishizawa T, Vahrmeijer AL, Mieog JSD (2018). A practical guide for the use of indocyanine green and methylene blue in fluorescence-guided abdominal surgery. J Surg Oncol.

[24] Marshall MV, Draney D, Sevick-Muraca EM, Olive DM (2010). Single-dose intravenous toxicity study of IRDye 800CW in Sprague-Dawley rats. Mol Imaging Biol.

[25] Rosenthal EL, Warram JM, De Boer E (2015). Safety and tumor specificity of cetuximab-IRDye800 for surgical navigation in head and neck cancer. Clin Cancer Res.

[26] Price EW, Orvig C (2014). Matching chelators to radiometal for radiopharmaceuticals. Chem Soc Rev.

