# Supplementary material for: In Vivo Evaluation of Indium-111–Labeled 800CW as a Necrosis-Avid Contrast Agent
Source: Mol Imaging Biol. 2020 Jun 8;22(5):1333–41. doi: 10.1007/s11307-020-01511-x (PMC7497446; doi:10.1007/s11307-020-01511-x)
Supplement: Supplementary file 1 — (DOCX 21292 kb) [file 11307_2020_1511_MOESM1_ESM.docx]

*In Vivo* Evaluation of Indium-111 Labeled 800CW as a Necrosis Avid Contrast Agent

**Marcus C.M. Stroet^1,2^, Erik de Blois^1^, Debra C. Stuurman^3^, Corrina M.A. de Ridder^1,3^, Joost Haeck^4^, Yann Seimbille^1^, Laura Mezzanotte^1,2^,** **Marion de Jong^1^, Clemens W.G.M. Löwik^1,2,5^, Kranthi M. Panth^1,2*^**

1. Erasmus MC, University Medical Center Rotterdam, Department of Radiology & Nuclear Medicine, Rotterdam.
2. Erasmus MC, University Medical Center Rotterdam, Department of Molecular Genetics, Rotterdam.
3. Erasmus MC, University Medical Center Rotterdam, Department of Urology, Rotterdam.
4. AMIE Core Facility, Erasmus MC, Rotterdam, the Netherlands
5. CHUV Department of Oncology, University of Lausanne, Switzerland

*. Corresponding author [k.panth@erasmusmc.nl](mailto:k.panth@erasmusmc.nl)

# Supporting information

## Supplement 1: Chromatogram DOTA-PEG_4_-800CW


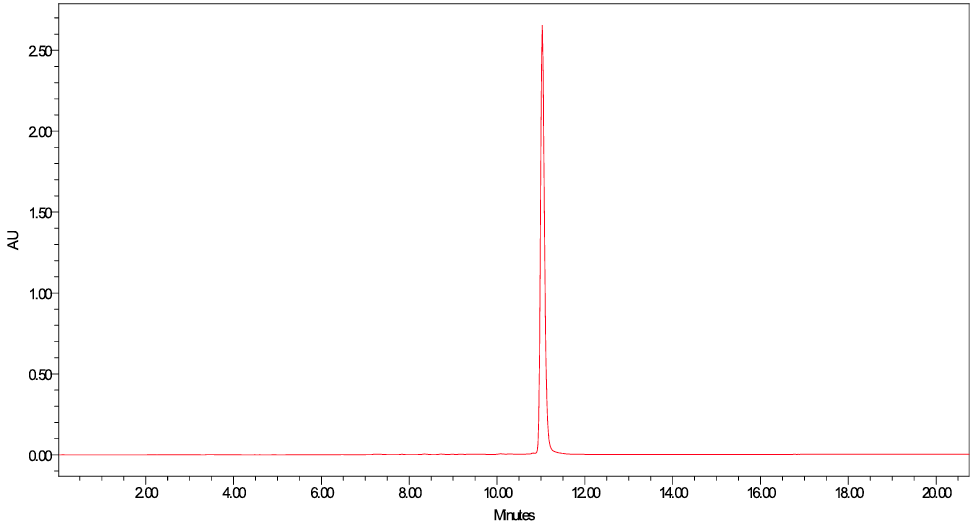


HPLC chromatogram (method B, measured at 254 nm) of the precursor **1** after purification. Precursor **1** eluted at 11.0 minutes and has a purity of >99%.

## Supplement 2: Chromatograms native indium labeling


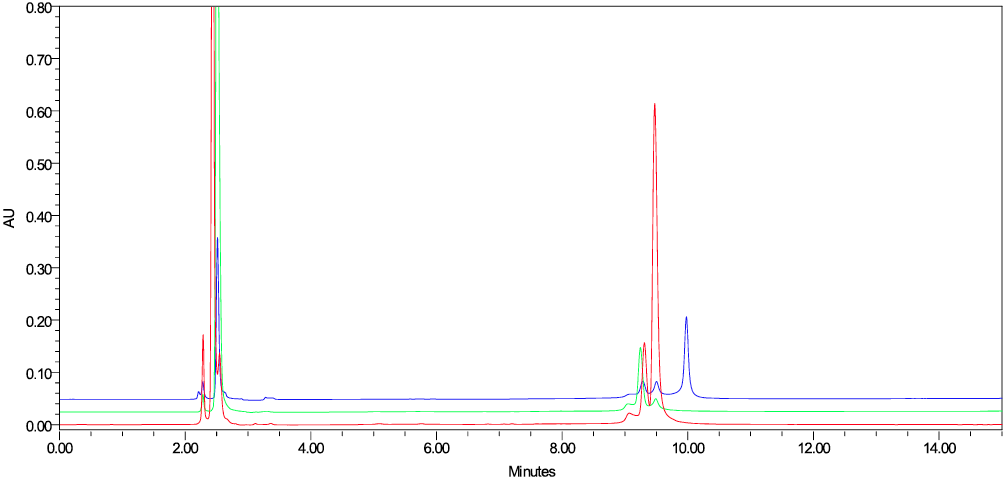


HPLC chromatograms (method B, measured at 254 nm). In red: In-DOTA-PEG_4_-800CW. The peak at 9.5 minutes was isolated and reinjected on mass spectrometry. From the mass we identified the peak at 9.5 min as In-DOTA-PEG_4_-800CW. ESI-MS: *m/z* calculated for C_72_H_100_N_8_O_25_S_4_In^+^ was 1719.47. *m/z* found was 860.19 [M+H]^2+^. In green: In-DOTA-PEG_4_-800CW + gentisic acid spike. The peak at 9.35 min increased and is therefore identified as gentisic acid. In blue: In-DOTA-PEG_4_-800CW + DOTA-PEG_4_-800CW spike. DOTA-PEG_4_-800CW eluted at 10.1 min, which is not observed in the HPLC trace of the reaction mixture, indicating full consumption of the precursor.

## Supplement 3: Stability [^111^In]In-DOTA-PEG_4_-800CW after two days at room temperature


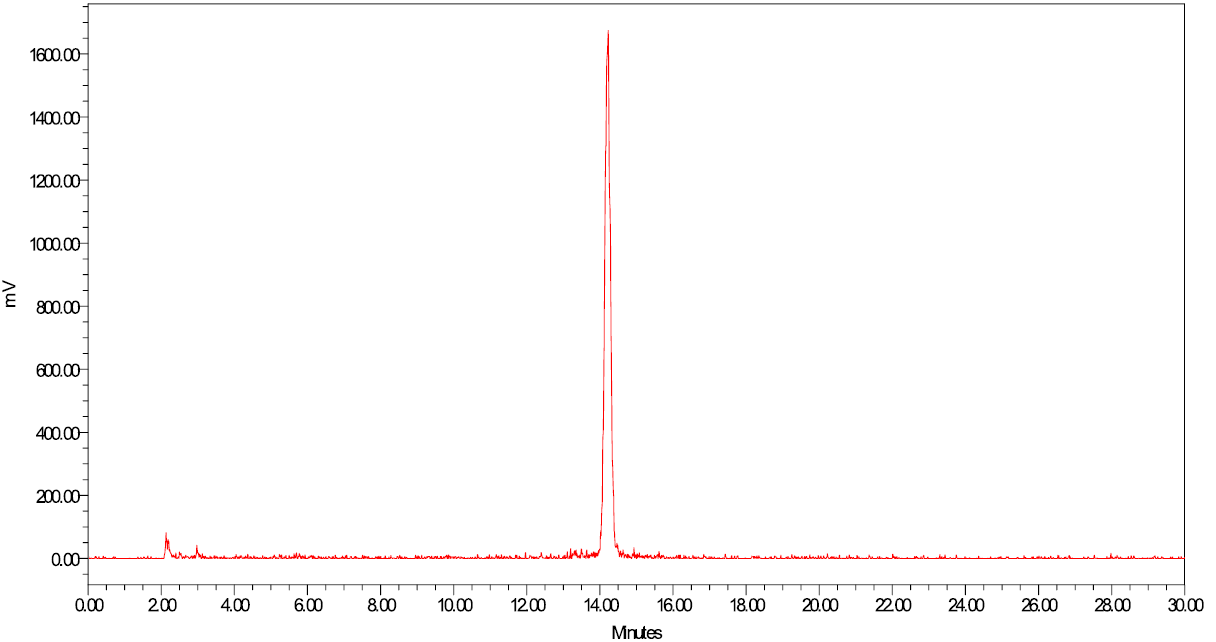


Radio-HPLC chromatogram (method C) of [^111^In]In-DOTA-PEG_4_-800CW (10 MBq/nmol in 200 µL).

## Supplement 4: BLI image tumor necrotic core


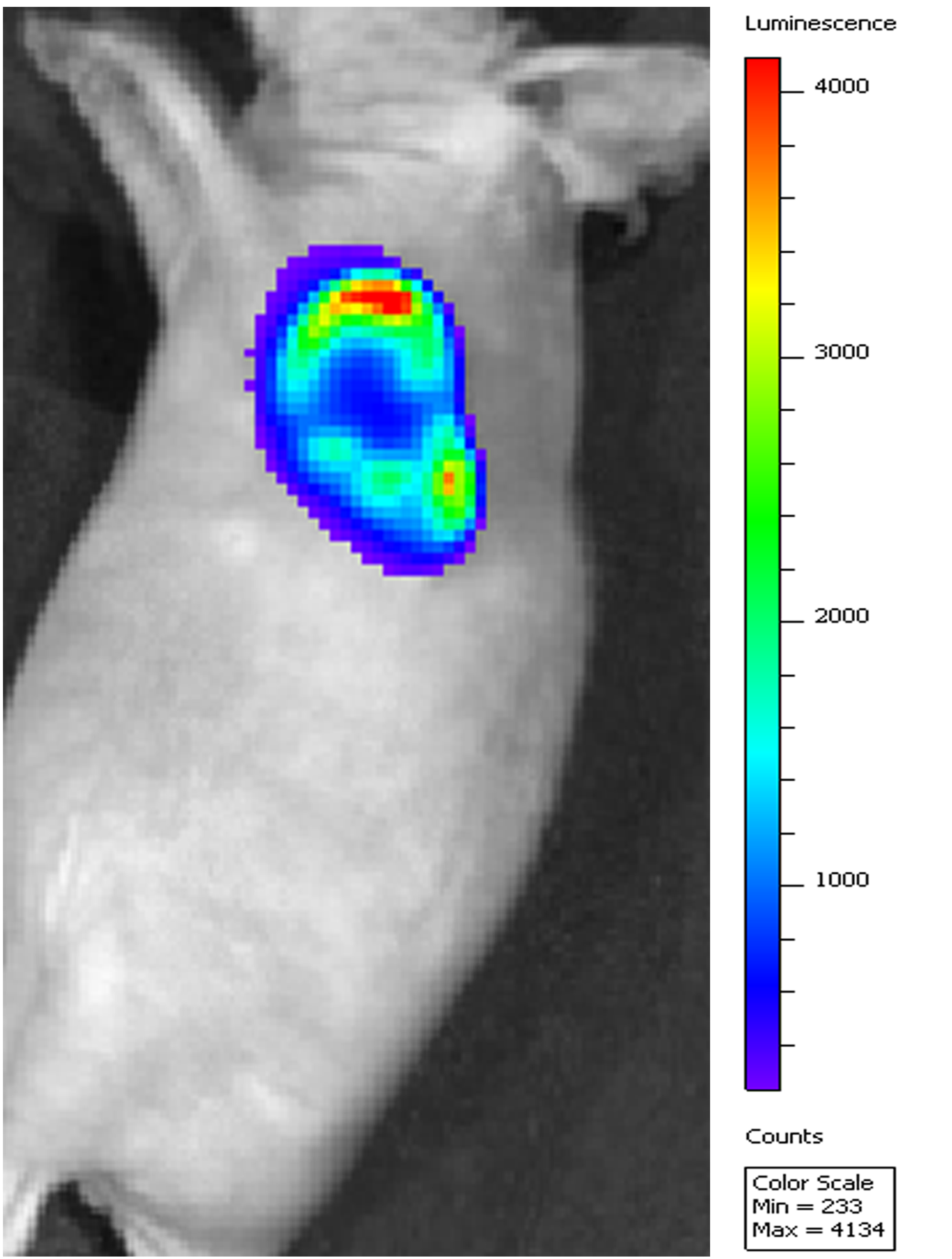


BLI image taken from mouse from a mouse, two weeks after 4T1-Luc2 cells were inoculated on the shoulder. The mice were injected with D-luciferin (150 mg/kg, i.p. injection). 10 min later, BLI was performed for 30 sec with an open filter under isoflurane anesthesia (4% induction, 1.5 to 2% maintenance in 100% O_2)_.

## Supplement 5: Tumor to blood ratio over time


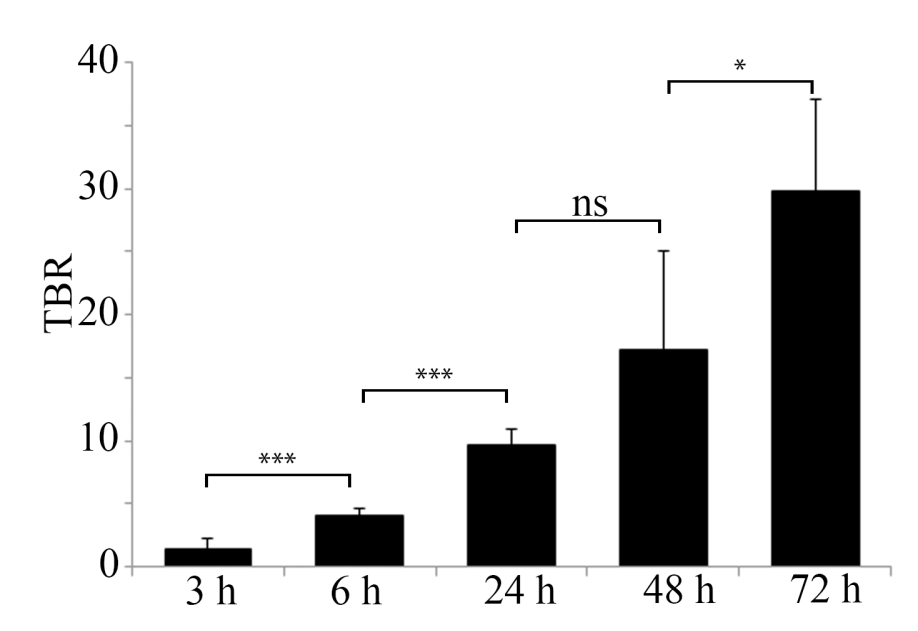


Tumor to background ratio (tumor to blood) over time from biodistribution experiments at medium (2 nmol), and low dose (0.2 nmol), n = 6. ns for no significant difference;

* for p<0.05; *** for p<0.005.

## Supplement 6: TACs dynamic scans

##
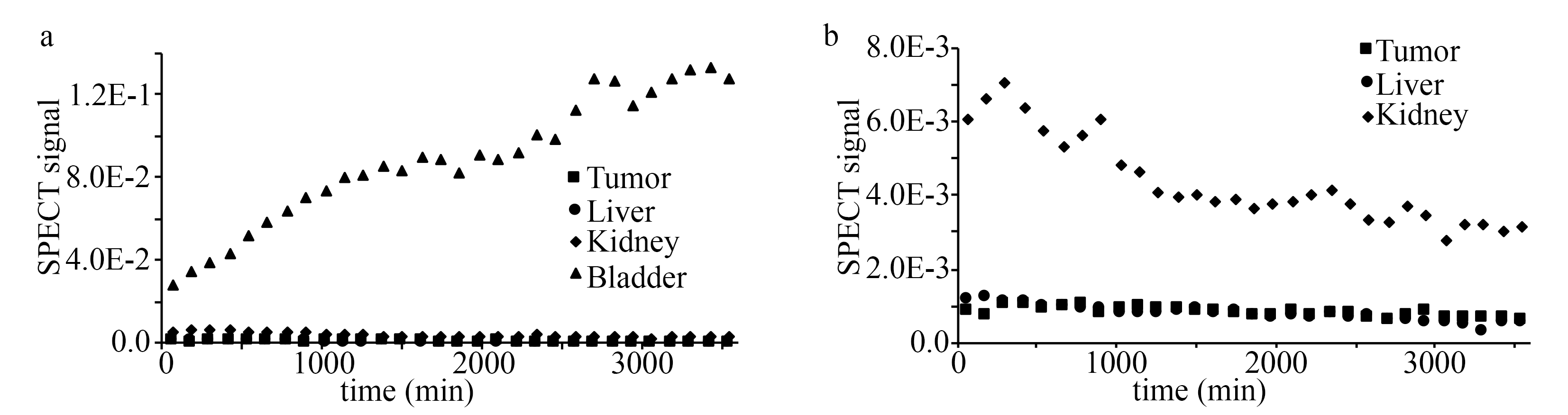


a) Time activity curves of bladder, kidney, liver, and tumor, obtained with a dynamic SPECT/CT scan over the first hour after injection of [^111^In]**1**. B) the same time activity curves as in (a) but excluding the bladder. From the TACs it can be concluded that there is a rapid clearance of [^111^In]**1** via the kidneys to the bladder.
